# Signs, symptoms and comorbidities of COVID-19 infection in informal workers in Medellin, Colombia, 2021

**DOI:** 10.47626/1679-4435-2025-1389

**Published:** 2025-11-04

**Authors:** María Osley Garzón-Duque, Ana Sofía Castaño Giraldo, Fabio León Rodríguez Ospina, Andrea Alexandra Boscan Sequera, Valeria Miranda Blandón, Javier Andrés Márquez Hernández, Osorio Jacobo Castaño

**Affiliations:** 1 Universidad CES, Facultad de Medicina, Medellín, Colombia; 2 Universidad de Antioquia, Facultad Nacional de Salud Pública, Medellín, Colombia

**Keywords:** COVID-19, informal sector, working conditions, health vulnerability, pandemics., COVID-19, sector informal, condiciones de trabajo, vulnerabilidad en salud, pandemias.

## Abstract

**Introduction:**

Despite numerous studies on COVID-19, there is still limited evidence regarding its behavior in subsistence workers.

**Objectives:**

To describe how sociodemographic conditions, signs, symptoms, and comorbidities are related to COVID-19 infection among informal workers in Medellin, Colombia, in 2021.

**Methods:**

This cross-sectional study used primary data obtained from a broader project. A total of 656 workers were recruited through snowball sampling. The study assessed sociodemographic variables, infection characteristics, signs, symptoms, and comorbidities. Univariate, bivariate and multivariate analyses were performed.

**Results:**

The prevalence of COVID-19 diagnosis was significantly higher (p < 0.05) among individuals who had contact with a probable or confirmed case, underwent testing due to contact with a confirmed case, had a coworker or family member with COVID-19, had contact with an infected family member and who did not receive medical care. The presence of fever, dry cough, dyspnea, myalgia, nausea and/or vomiting, abdominal pain, ageusia/dysgeusia, anosmia, consultation for infection symptoms, COVID-19 testing, and previous diagnosis of diabetes were also associated with COVID-19. Contact with an infected family member and who did not receive medical care, as well as consultation for infection symptoms also explained a higher rate of COVID-19 diagnosis.

**Conclusions:**

During the pandemic, confinement and cohabitation conditions may have facilitated the spread of infection, increasing the socio-labor and environmental vulnerability of the working population under study.

## Abstract (es)

**Introducción:**

Aunque se han realizado múltiples estudios sobre COVID-19, aún existe poca evidencia acerca de su comportamiento en trabajadores con empleos de subsistencia.

**Objetivos:**

Describir la relación entre las condiciones sociodemográficas, los signos, síntomas y comorbilidades, y la infección por COVID-19 en trabajadores informales en Medellín, Colombia, 2021.

**Métodos:**

Estudio transversal con fuente primaria de información derivado de un macroproyecto. Se encuestaron a 656 trabajadores, seleccionados mediante muestreo tipo bola de nieve. Se evaluaron variables sociodemográficas, características de la infección, signos, síntomas y comorbilidades. Se realizaron análisis univariados, bivariados y multivariados.

**Resultados:**

La prevalencia del diagnóstico de COVID-19 fue mayor (p < 0,05) en quienes tuvieron contacto con caso probable o confirmado, se les realizaron prueba por contacto con caso confirmado, tuvieron compañero de trabajo o familiar con COVID-19, contacto con familiar infectado y que no recibió atención médica. La presencia de fiebre, tos seca, disnea, mialgias, nauseas y/o vómito, dolor abdominal, aneugesia/disgeusia anosmia, consulta por síntomas, realización de prueba para COVID-19 y diagnóstico previo de diabetes también se asociaron con la infección por COVID-19. En el modelo ajustado, contacto con familiar infectado o con familiar que no recibió atención médica y consulta por síntomas explicaron el incremento en el diagnóstico de COVID-19.

**Conclusiones:**

Durante la pandemia, las condiciones de confinamiento y convivencia pudieron haber favorecido la propagación de la infección, incrementando la vulnerabilidad sociolaboral y ambiental de la población trabajadora en estudio.

## INTRODUCTION

COVID-19 is a respiratory viral disease caused by SARS-CoV-2 whose clinical course may vary from mild to moderate. Common symptoms include fever, cough, fatigue, ageusia, and anosmia; in severe cases, patients may present with severe dyspnea or chest pain.^1^

The 2020 COVID-19 pandemic had a profound impact on the health of the global population. Individuals, regardless of background, experienced either acute or chronic effects on their baseline health. In order to better understand the repercussions and sequelae of COVID-19 in subsistence workers, it is also necessary to study and analyze the individual manifestations of the infection.

Globally, informal work represents one of the main sources of employment and has risen significantly in developing countries, reaching figures as high as 35-90%.^2^ Subsistence workers face greater vulnerability, especially those who use streets and sidewalks as their workplace. With very limited job opportunities and few possessions, they are more likely to be affected in critical situations, such as the 2020 pandemic.^3^

During the pandemic, misinformation and barriers to vaccination among low-income and homeless populations emerged as major challenges, as concerns about side effects led vaccination refusal rates between 35.7% and 48.0%. Reasons for delaying or rejecting vaccination are complex and multifaceted, including convenience, availability and access, understanding and clarity of health information, judgments about risks and benefits, perceptions of collective versus individual responsibility, trust or distrust in medical care, beliefs, traditions, or ideologies, all compounded by low levels of health literacy. Promotion of health equity and reduction of morbidity and mortality require educating the population about vaccination.^4^,^5^

Immigrants, unemployed individuals, those with low educational levels, inadequate housing conditions, or living in overcrowded setting faced an increased risk of death from COVID-19. Similarly, residents of urban areas characterized by higher poverty rates, rental housing, lack of insurance, and overcrowded households showed a greater likelihood of testing positive for COVID-19. These findings underscore the association between infection transmission and individuals’ socioeconomic backgrounds and characteristics.^6^

The present study focuses on subsistence workers affected by the COVID-19 pandemic. Data from the Internation Labor Organization (ILO) indicate that, as of 2018, 53.1% of the working population in Latin America and the Caribbean was employed in the informal sector,^7^ with limited access to health care services and exposure to social inequalities. Within this framework, the workers participating in this study exhibit occupational risk factors that increase their vulnerability during health crisis such as the COVID-19 pandemic. Therefore, this study aims to describe the conditions under which COVID-19 infection occurred among informal workers from Medellín, Colombia, in 2021, in order to identify the relationship between infection characteristics, signs and symptoms, comorbidities, and sociodemographic conditions.

## METHODS

### DESIGN

This is a cross-sectional study with an analytical approach and primary sources of information that stems from the broader project titled “Living, working, and health conditions among informal street vendors in Medellín during and after the pandemic (2021-2022),” approved by the Institutional Human Ethics Committee of Universidad CES, under record no. 156, of January 27, 2021.

### POPULATION

Informal street vendors (*venteros*) from Medellín and the district (*corregimiento*) of San Antonio de Prado were reached at their sales posts, meetings, and guild assemblies, through their leaders and the principal investigator. A group of leaders and workers contributed to the planning and implementation of the fieldwork, as part of a collaborative knowledge-generation process that has been in development for over 19 years. Data collection was carried out using assisted surveys at locations agreed upon with leaders and workers, employing convenience sampling with a snowball technique, for a total of 656 workers surveyed between March and November 2021.

### INCLUSION CRITERIA

Eligible workers were individuals > 18 years with ≥ 3 years in their trade, which ensured that they had been engaged in their occupation prior to the pandemic and had not adopted it as an emergency measure after losing employment in other jobs. In addition, participants were required to be informed about the study, its procedures, benefits, scopes, and limitations, and had to agree to participate. No participants were excluded based on the established criteria (leaving their trade ≥ 1 year and failure to sign informed consent prior to data collection).

### VARIABLES

The dependent variable was COVID-19 infection (1. Yes; 2. No), diagnosed and self-reported by workers at the time of data collection. The independent variables encompassed: 1) sociodemographic conditions; sex, age (recategorized into four groups: 18-29; 30-44; 45-49; ≥ 60 years), health insurance coverage, type and condition of housing, cohabitation with other families, and patterns of housing use; 2) infection-related characteristics at the individual, family, and workplace levels: contact with a probable case, testing due to contact with a confirmed case, turnaround time for test results, reasons for not testing, management of the confirmed case, vaccination status, type of vaccine received, history of infection in co-workers, contact with infected co-workers, clinical course of infected co-workers, reason for suspecting infection in a co-worker, history of infection in family members, contact with infected family members, relationship to the infected family member, clinical course of infected family members, reasons for suspecting infection in a family member; c) additional variables: infection-related signs and symptoms, reasons for not being tested in the past year, comorbidities such as hypertension, diabetes, chronic obstructive pulmonary disease (COPD), obesity, and depressive symptoms.

### MINIMIZATION OR ERRORS AND BIASES

Statistical tests consistent with the nature and level of measurement of the variables were applied, along with the epidemiological measures corresponding to this type of study design. Selection bias was minimized through the strict application of inclusion criteria and the use of convenience snowball sampling method, which helped represent certain characteristics of the study population. Although inference error could not be estimated, this approach is more suitable than a non-representative sampling strategy, which can only be applied to the data collection sample itself. Information bias was controlled by standardizing the research team and interviewer, conducting a pilot test, and administering an assisted survey that was validated for form and content with both worker leaders and participating worker.

### ANALYSIS

Descriptive, bivariate, and multivariate analyses were performed to explore non-causal associations and relationships between explanatory variables and COVID-19 infection among workers who reported having been diagnosed with this disease. Absolute and relative frequencies were calculated for the dependent variable (COVID-19; 1. Yes; 2. No) and all independent variables. For the bivariate analysis, chi-square association tests were applied along with the estimation of prevalence ratios (PR) and their 95%CI. The multivariate analysis used binary logistic regression to identify factors or conditions contributing to the presence of COVID-19 among workers. Independent variables were entered sequentially, from lowest to highest p-value, based on the bivariate analysis results, in accordance with the Hosmen Lemenchow criterion (p < 0.25).

Statistical analyses were conducted with a 95%CI and a 5.0% margin of error. Calculations were carried out using SPSS^®^, version 26, licensed to Universidad de Antioquia, and Epidat version 3.1. Tables and texts were prepared in Excel and Word.

## RESULTS

**Figure 1:**
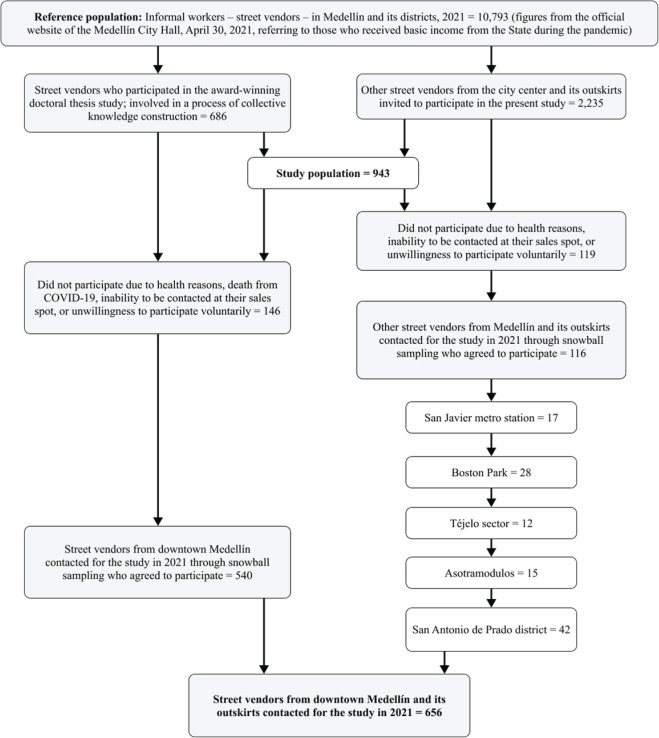
Selection of study population.

The study participant selection flow appears in Figure 1.

### SOCIODEMOGRAPHIC CONDITIONS AND COVID-19 INFECTION CHARACTERISTICS AMONG WORKERS, THEIR COWORKERS, AND THEIR FAMILY MEMBERS

The majority of workers were aged 45 years or older (74.7%), particularly men aged ≥ 60 years (38.7%). Among women, 28.0% were between 30 and 44 years old. Over 97.0%, had health insurance coverage (Table 1).

**Table 1:** Frequency and percentage distribution of sociodemographic conditions and COVID-19 infection characteristics by biological sex among the working population under study, Medellín, Colombia, 2021 (n = 656)

Characteristic/condition	Biological sex		Total n (%)
	Men	Women	
	n (%)	n (%)	
Demographic conditions			
Age (years) (n = 656)			
18-29	10 (2.7)	14 (4.9)	24 (3.7)
30-44	62 (16.8)	80 (28.0)	142 (21.7)
45-59	155 (42.0)	127 (44.4)	282 (43.0)
≥ 60	143 (38.7)	65 (22.7)	208 (31.7)
Health insurance coverage (n = 656)			
Yes	358 (97.0)	278 (97.2)	636 (97.0)
No	12 (3.2)	8 (2.8)	20 (3.0)
Type of housing (n = 656)			
House	203 (55.0)	140 (49.0)	343 (52.3)
Apartment	113 (30.5)	116 (40.6)	229 (35.0)
Room	27 (7.3)	13 (4.6)	40 (6.1)
Tenancy	15 (4.0)	11 (3.9)	26 (4.0)
Other	12 (3.2)	6 (2.1)	18 (2.7)
Condition of housing (n = 656)			
Good	231 (62.4)	182 (63.6)	413 (63.0)
Fair	132 (36.0)	94 (33.0)	226 (34.5)
Poor	7 (1.9)	10 (3.5)	17 (2.6)
Cohabitation with other families (n = 656)			
Yes	59 (16.0)	40 (14.0)	99 (15.1)
No	311 (84.0)	246 (86.0)	557 (84.9)
Pattern of housing use (n = 656)			
Residencial	365 (98.7)	283 (99.0)	648 (98.8)
Mixed	5 (1.4)	3 (1.0)	8 (1.2)
Probable and confirmed cases of COVD-19			
Contact with a probable or confirmed case (n = 654)			
Yes	78 (21.1)	86 (30.1)	164 (25.0)
No	290 (78.9)	200 (69.9)	490 (74.7)
Testing due to contact with a confirmed case (n = 119)			
Yes		25 (42.4)	55 (46.2)
No	30 (50.0)	34 (57.6)	64 (53.8)
Turnaround time for test results (days) (n = 117)			
0	1 (1.7)	1 (1.7)	2 (1.7)
1-3	55 (93.2)	51 (87.9)	106 (90.6)
4-10	2 (3.4)	5 (8.6)	7 (6.0)
> 10	1 (1.7)	1 (1.7)	2 (1.7)
Result for the test (n = 118)			
Positive	25 (41.7)	26 (44.8)	51 (43.2)
Negative	33 (55.0)	31 (53.4)	64 (54.0)
Did not receive	2 (3.3)	1 (1.7)	3 (2.5)
Positive test - received medical care (n = 51)			
Yes	23 (95.8)	22 (81.4)	45 (88.2)
No	1 (4.2)	5 (18.6)	6 (11.8)
Reasons for not testing			
Did not present symptoms (n = 213)			
Yes	28 (24.8)	35 (35.0)	63 (30.0)
No	85 (75.2)	65 (65.0)	150 (70.0)
Symptoms lasted for less than 1 week (n = 213)			
Yes	81 (71.7)	57 (57.0)	138 (64.8)
No	32 (28.3)	43 (43.0)	75 (25.2)
Dis not visited a physician in the past year (n = 213)			
Yes	2 (14.3)	6 (6.0)	8 (3.7)
No	11 (85.7)	94 (94.0)	205 (96.3)
Knew where to obtain a diagnostic test (n = 212)			
Yes	1 (0.9)	0 (0.0)	1 (0.5)
No	112 (99.1)	99 (100.0)	211 (99.5)
Symptomatic, without having been tested (n = 210)			
Yes	1 (0.9)	3 (3.1)	4 (1.9)
No	112 (99.1)	94 (96.9)	206 (98.1)
Some family member had COVID-19 in the past year (n = 656)			
Yes	162 (43.8)	143 (50.0)	305 (46.5)
No	208 (56.2)	143 (50.0)	351 (53.5)
Infection in workers, coworkers, and family members			
Management of confirmed cases of COVID-19 (n = 47)			
Treated at home	13 (56.5)	7 (29.2)	20 (42.6)
Treated at home with the COVID-19 kit	0 (0.0)	5 (20.8)	5 (10.6)
Medical follow-up	4 (17.4)	5 (20.8)	9 (19.1)
Received home-based hospital care	0 (0.0)	0 (0.0)	0 (0.0)
Required hospitalization	6 (26.1)	7 (29.2)	13 (27.7)
Hospitalization involving in-hospital transfer (n = 6)			
Special care unit	2 (66.7)	0 (0.0)	2 (33.3)
Intensive care unit	1 (33.3)	3 (100.0)	4 (66.7)
Vaccination status at the time of the survey (n = 647)	/		
Yes	272 (74.9)	210 (73.9)	482 (74.5)
No	91 (25.1)	74 (26.1)	165 (25.5)
Vaccine received (n = 481)			
Sinovac	56 (20.7)	45 (21.3)	101 (21.0)
Janssen	30 (11.1)	38 (18.0)	68 (14.1)
Pfizer	102 (37.7)	78 (37.0)	180 (37.4)
AstraZeneca	54 (20.0)	23 (10.9)	77 (16.0)
Moderna	28 (10.4)	27 (12.8)	55 (11.4)
Coworker had COVID-19 in the past year (n = 656)			
Yes	137 (37.0)	132 (46.2)	269 (41.0)
No	233 (63.0)	154 (53.8)	387 (59.0)
Coworker was diagnosed with COVID-19 in the past year (n = 269)			
Yes	93 (63.3)	111 (84.1)	204 (75.8)
No	44 (32.7)	21 (15.9)	65 (24.2)
Clinical course of coworker with COVID-19 (n = 268)			
Did not receive medical care	2 (1.50)	2 (1.52)	4 (1.50)
Treated at home	70 (51.5)	60 (45.5)	130 (48.5)
Received home-based hospital care	2 (1.50)	1 (0.76)	3 (1.12)
Resorted to self-medication	2 (1.50)	0 (0.0)	2 (0.80)
Visited a physician	33 (24.3)	31 (23.5)	64 (23.9)
Required hospitalization	27 (19.9)	38 (28.8)	65 (24.3)
Reason to consider that the coworker was infected with COVID-19 (n = 106)			
A contact was infected	1 (1.50)	0 (0.0)	1 (0.9)
Another coworker reported it	2 (2.9)	1 (2.6)	3 (2.8)
Coworker presented COVID-19 symptoms	60 (88.2)	31 (81.6)	91 (85.8)
The coworker him/herself reported it	5 (7.4)	5 (13.2)	10 (9.4)
Coworker’s leader reported it	0	1 (2.6)	1 (0.9)
Relationship with the infected family member (n = 305)			
Parent	9 (5.6)	16 (11.2)	25 (8.2)
Sibling	54 (33.3)	41 (28.7)	95 (31.2)
Child	30 (18.5)	45 (31.5)	75 (24.6)
Grandchild	0 (0.0)	1 (0.70)	1 (0.33)
Other	69 (42.6)	40 (28.0)	109 (35.7)
Contact with the infected family member (n = 305)			
Yes	54 (33.3)	56 (39.2)	110 (36.1)
No	108 (66.7)	87 (60.8)	195 (63.9)
Family member diagnosed with COVID-19 (n = 305)			
Yes	142 (87.7)	121 (84.6)	263 (86.2)
No	20 (12.3)	22 (15.4)	42 (13.8)
Clinical course of family member with COVID-19 (n = 305)			
Did not receive medical care	8 (4.9)	5 (3.50)	13 (4.3)
Treated at home	98 (60.5)	94 (65.7)	192 (62.9)
Received home-based hospital care	9 (5.6)	9 (6.3)	18 (5.90)
Resorted to self-medication	0 (0.0)	3 (2.10)	3 (1.0)
Visited a physician	1 (0.62)	0 (0.0)	1 (0.33)
Required hospitalization	29 (17.9)	13 (9.1)	42 (13.8)
Died	17 (10.5)	19 (13.3)	36 (11.8)
Family member was infected with COVID-19 but was not tested (n = 55)			
A contact was infected	1 (3.3)	1 (4.0)	2 (3.6)
Family member presented COVID symptoms	29 (96.7)	24 (96.0)	53 (96.4)

The majority of men and women lived in houses or apartments (87.3%). Overall, 34.5% described their housing condition as fair and 2.6%, as poor. Furthermore, 15 out of every 100 workers lived with other families, and 98.7% used their home exclusively for residential purposes (Table 1).

### PROBABLE AND CONFIRMED CASES OF COVID-19

Overall, 25.0% (n = 164) of participants reported having had contact with a probable or confirmed case of COVID-19 during the pandemic, a situation more frequent among women. Of those reporting contact with a confirmed case, 46.2% (n = 55) underwent diagnostic testing, with men accounting for a larger proportion. Among those tested, 90.6% received results within 1 to 3 days; 43.2% (n = 51) tested positive, more commonly women, whereas 2.5% never received their results. Finally, 88.2% of those who tested positive received medical care, predominantly men (95.8%) (Table 1).

Among those diagnosed with COVID-19, 42.6% (n = 20) received medical care at home; 10.6% managed their condition at home with a COVID-19 kit; 19.1% received medical follow-up; and 27.7% required hospitalization. Of those hospitalized, 66.7% (n = 4) were transferred to the intensive care unit (ICU) and 33.3% (n = 2) to the special care unit (SCU), with three women being admitted to the ICU and two men to the SCU (Table 1).

### VACCINATION STATUS

A total of 74.5% reported being vaccinated, with similar proportions between men and women. Of these, 37.4% (n = 180) received the Pfizer vaccine, 21.0% (n = 101) Sinovac, 16.0% (n = 77) AstraZeneca, 14.1% (n = 68) Janssen, and 11.4% (n = 55) Moderna (Table 1).

### REASONS FOR NOT UNDERGOING COVID-19 TESTING

A total of 70.0% (n = 150) of participants did not undergo COVID-19 testing because they were asymptomatic. However, 64.8% (n = 138) of symptomatic workers experienced symptoms for less than 1 week and were not tested, whereas 1.9% of those reporting symptoms did not undergo diagnostic testing. Furthermore, 99.5% did not know where to obtain a diagnostic test, 96.3% (n = 205) had not visited a physician in the past year, with this proportion being higher among women (94.0%; n = 94) (Table 1).

### FAMILY MEMBER WITH COVID-19

Overall, 46.5% (n = 305) of workers reported that a family member had contracted COVID-19, with similar rates for men and women. Moreover, 86.2% (n = 263) of family members had a confirmed diagnosis, more often among men (87.7%). Of these, 62.9% (n = 192) were treated at home, 13.8% (n = 42) required hospitalization, and 11.8% (n = 36) died. For family members without test confirmation, 96.4% (n = 53) reported experiencing the illness, and 3.6% (n = 2) reported contact with an infected person. Finally, 36.1% (n = 110) reported contact with an infected family member, especially women (39.2%). The infected family members were most often siblings (31.2%; n = 95) and children (24.6%; n = 75) (Table 1).

### COWORKER WITH A CLINICAL PRESENTATION OF COVID-19

Off all workers, 41.0% (n = 269) reported that a coworker had been infected with COVID-19 in the past year, more frequently among women (46.2%; n = 132). Of these, 75.8% (n = 204) received a confirmed COVID-19 diagnosis, again mostly women (84.1%; n = 111). Among diagnosed cases, 48.5% (n = 130) were treated at home, 23.9% (n = 64) consulted a physician, 24.3% (n = 65) required hospitalization, and 1.50% (n = 4) received no care. For those without test confirmation but who considered themselves infected, 85.5% (n = 91) reported COVID-19 symptoms, and 9.4% (n = 10) of coworkers reported being infected, with a higher prevalence among women (13.2%; n = 5) (Table 1).

### COVID-19-RELATED SIGNS AND SYMPTOMS REPORTED BY THE WORKING POPULATION UNDER STUDY

Results showed that 53.2% (n = 158) of the study population experienced fever > 38 ºC, more common among men (55.6%; n = 89). Dry cough was present in 27.3% (n = 81), with similar proportions across sexes. Dyspnea was reported by 17.9% (n = 53), and myalgia by 53.2% (n = 158), the latter more frequent among women (54.0%; n = 74). Ageusia was reported by 37.7%, particularly women (41.6%; n = 57); anosmia by 36.7% (n = 109); and headache by 40.1% (n = 119). Among symptomatic individuals, 34.5% visited a physician. Of those tested, 47.5% (n = 57) had experienced suggestive symptoms in the previous year (Table 2).

**Table 2:** Distribution of frequencies and percentages of COVID-19-related signs, symptoms and comorbidities reported by the working population under study (n = 656)

Characteristic/condition	Biological sex		Total n (%)
	Men	Women	
	n (%)	n (%)	
COVID-19 symptoms in the past year			
Fever > 38 ºC (n = 297)			
Yes	89 (55.6)	69 (50.4)	158 (53.2)
No	71 (44.4)	68 (49.6)	139 (46.8)
Dry cough (n = 297)			
Yes	44 (27.5)	37 (27.0)	81 (27.3)
No	116 (72.5)	100 (73.0)	216 (72.7)
Dyspnea (n = 297)			
Yes	27 (16.9)	26 (19.0)	53 (17.9)
No	133 (83.1)	111 (81.0)	244 (82.1)
Productive cough (n = 297)			
Yes	3 (1.9)	0 (0.0)	3 (1.0)
No	157 (98.1)	137 (100.0)	294 (99.0)
Sore throat (n = 297)			
Yes	15 (9.4)	13 (9.5)	28 (9.4)
No	145 (90.6)	124 (09.5)	269 (90.6)
Hemoptysis (n = 297)			
Yes	1 (0.6)	0 (0.0)	1 (0.3)
No	159 (99.4)	137 (100.0)	296 (99.7)
Confusion (n = 296)			
Yes	1 (0.6)	0 (0.0)	1 (0.3)
No	158 (99.4)	137 (100.0)	295 (99.7)
Myalgia (n = 297)			
Yes	84 (52.5)	74 (54.0)	158 (53.2)
No	76 (47.5)	63 (46.0)	139 (46.8)
Adynamia (n = 297)			
Yes	16 (10.0)	9 (6.6)	25 (8.4)
No	144 (90.0)	128 (93.4)	272 (91.6)
Headache (n = 297)			
Yes	59 (36.9)	60 (43.8)	119 (40.1)
No	101 (63.1)	77 (56.2)	178 (59.9)
Rhinorrhea (n = 297)			
Yes	1 (0.6)	1 (0.7)	2 (0.7)
No	159 (99.4)	136 (99.3)	295 (99.3)
Chest pain (n = 297)			
Yes	8 (5.0)	6 (4.4)	14 (4.7)
No	152 (95.0)	131 (95.6)	283 (95.3)
COVID-19 symptoms in the past year			
Underwent COVID-19 testing in the past year (n = 652)			
Yes	61 (16.2)	59 (20.7)	120 (18.4)
No	306 (83.4)	226 (79.3)	532 (81.6)
Reason for not visiting a physician due to COVID-19 symptoms			
Resorted to self-medication (n = 124)			
Yes	37 (51.4)	24 (46.2)	61 (49.2)
No	35 (48.6)	28 (53.8)	63 (50.8)
Fear (n = 124)			
Yes	5 (6.9)	3 (5.8)	8 (6.5)
No	67 (93.1)	49 (94.2)	116 (93.5)
Comorbidities (n = 656)			
Diabetes			
Yes	34 (9.2)	34 (11.9)	68 (10.4)
No	336 (91.2)	252 (88.1)	588 (89.6)
Hypertension			
Yes	90 (24.3)	69 (24.1)	159 (24.2)
No	280 (75.7)	217 (75.9)	497 (75.8)
COVID-19 in the past year			
Diarrhea (n = 297)			
Yes	30 (18.7)	23 (16.8)	53 (17.8)
No	130 (81.3)	114 (83.29	244 (82.2)
Nausea and/or vomiting (n = 297)			
Yes	15 (9.4)	17 (12.4)	32 (10.8)
No	145 (90.6)	120 (87.6)	265 (89.2)
Abdominal pain (n = 296)			
Yes	10 (6.3)	4 (2.9)	14 (4.7)
No	149 (93.7)	133 (97.1)	282 (95.3)
Ageusia/dysgeusia (n = 297)			
Yes	55 (34.4)	57 (41.6)	112 (37.7)
No	105 (65.6)	80 (58.4)	185 (62.3)
Anosmia (n = 297)			
Yes	55 (34.4)	54 (39.4)	109 (36.7)
No	105 (65.6)	83 (60.6)	188 (63.3)
No COVID-19 symptoms in the past year (n = 296)			
Yes	49 (30.8)	43 (31.4)	92 (31.1)
No	110 (69.2)	94 (68.6)	204 (68.9)
Visited a physician for any of the previous symptoms (n = 206)			
Yes	36 (31.6)	35 (38.0)	71 (34.5)
No	78 (68.4)	57 (62.0)	135 (65.5)
Site of consultation for COVID-19 symptoms			
Hospital (n = 90)			
Yes	11 (23.4)	9 (20.9)	20 (22.2)
No	36 (76.6)	34 (79.1)	70 (77.8)
Health post or health center (n = 86)			
Yes	6 (13.3)	9 (22.0)	15 (17.4)
No	39 (86.7)	32 (78.0)	71 (82.6)
Private physician (n = 87)			
Yes	4 (8.9)	4 (9.5)	8 (9.2)
No	41 (91.1)	38 (90.5)	79 (90.8)
Another location (n = 87			
Yes	14 (31.1)	15 (35.7)	29 (33.3)
No	31 (68.9)	27 (64.3)	58 (66.7)
Underwent testing for any of the symptoms (n = 120)			
Yes	29 (47.5)	28 (47.5)	57 (47.5)
No	32 (52.5)	31 (52.5)	63 (52.5)
Reason for not testing in the past year			
Did not consider it necessary (n = 124)			
Yes	1 (1.4)	3 (5.8)	4 (3.2)
No	71 (98.6)	49 (94.2)	120 (96.8)
Other (n = 124)			
Yes	49 (68.1)	36 (69.2)	85 (68.5)
No	23 (31.9)	16 (30.8)	39 (31.5)
Comorbidities (n = 656)			
Chronic obstructive pulmonary disease			
Yes	7 (1.9)	5 (1.7)	12 (1.8)
No	363 (98.1)	281 (98.3)	644 (98.2)
Obesity			
Yes	64 (17.3)	116 (40.6)	180 (27.4)
No	306 (82.47)	170 (50.4)	476 (72.6)
Depressive symptoms			
Moderate/severe	16 (4.3)	12 (3.1)	28 (4.3)
Absent/mild	354 (95.7)	274 (96.9)	628 (95.7)

It was found that 22.2% (n = 20) sought medical care at a hospital, 17.4% (n = 15) at a health post or health center, 9.2% (n = 8) with a private physician, and 33.3% (n = 29) at another location. Overall, 65.5% (n = 135) experienced symptoms but did not visit a physician, a situation more frequent among men (68.4%; n = 78). The main reasons for not seeking care included self-medication (49.2%; n = 61), perceiving it as unnecessary (3.2%; n = 4), and fear (6.5%; n = 8) (Table 2).

### SOCIODEMOGRAPHIC CONDITIONS AND INFECTION CHARACTERISTICS ASSOCIATED WITH COVID-19 DIAGNOSIS IN THE WORKING POPULATION UNDER STUDY

Significant associations (p < 0.05) were observed the prevalence of infection and certain characteristics in workers, their coworkers, and family members. COVID-19 diagnosis was 2.23 times more common among workers who had contact with a probable or confirmed case than in those who did not report such contact (PR = 3.23; 95%CI = 1.95-5.47). Additionally, the prevalence of COVID-19 was 61.0% greater among those required to undergo testing due to contact with a confirmed case, compared to those without such contact (PR = 1.61; 95%CI = 1.04-2.47) (Table 3).

**Table 3:** Sociodemographic conditions and infection characteristics associated with COVID-19 diagnosis in the working population under study, Medellín, Colombia, 2021 (n = 656)

Characteristic - condition	COVID-19		Total n (%)	Chi-square (p-value)^*^	Prevalence ratio (95% CI)
	Yes n (%)	No n (%)			
Sociodemographic conditions					
Biological sex (n = 656)					
Mele	25 (6.8)	345 (93.2)	370 (56.4)	0.90 (0.342)	0.77 (0.45;1.32)
Female	25 (8.7)	261 (91.3)	286 (45.6)		1.00
Age (years) (n = 656)					
18-29	2 (8.3)	22 (91.7)	24 (3.7)	0.08 (0.771)	1.00
30-44	10 (7.0)	132 (93.0)	142 (21.6)		0.84 (0.19;3.62)
45-59	21 (7.4)	261 (92.6)	282 (39.9)		0.89 (0.22;3.58)
≥ 60	17 (8.2)	191 (91.8)	208 (31.7)		0.98 (0.24;3.99)
Cohabitation with other family (n = 656)					
Yes	11 (11.1)	88 (88.9)	99 (15.1)	2.02 (0.155)	1.58 (0.84;2.99)
No	39 (7.0)	518 (93.0)	557(84.9)		1.0
Contact with a probable or confirmed case of COVID-19 (n = 654)					
Yes	26 (15.9)	138 (84.1)	164 (25.1)	20.8 (0.000)	3.23 (1.91;5.47)
No	24 (4.9)	466 (95.1)	490 (74.9)		1.0
Testing due to contact with a confirmed case (n = 119)					
Yes	29 (52.7)	26(47.3)	55 (46.2)	4.81 (0.028)	1.61 (1.04;2.47)
No	21(32.8)	43(67.2)	64 (53.8)		1.0
Positive test - received medical care (n = 51)					
Yes	44 (97.8)	1 (2.2)	45 (88.2)	2.93 (0.086)	1.17 (0.81;1.68)
No	5 (83.3)	1(16.7)	6 (11.8)		1.0
Vaccination status at the time of the survey (n = 647)					
Yes	38 (7.9)	444 (92.1)	482 (74.5)	0.06 (0.799)	1.08 (0.58;2.02)
No	12 (7.3)	153 (92.7)	165 (25.5)		1.00
Vaccine received (n = 481)					
Sinovac	8 (7.9)	93 (92.1)	101 (21.0)	6.46 (0.167)	0.95 (0.42;2.16)
Janssen	7 (10.3)	61 (89.7)	68 (14.1)		1.24 (0.53;2.90)
AstraZeneca	2 (2.6)	75 (97.4)	77 (16.0)		0.31 (0.07;1.33)
Moderna	8 (14.5)	47 (85.5)	55 (11.4)		1.75 (0.78;3.90)
Pfizer	15 (8.3)	165 (91.7)	180 (37.4)		1.00
Reasons for not testing in the past year (n = 213)					
Did not present symptoms					
Yes	2 (3.2)	61 (96.8)	63 (29.6)	0.10 (0.745)	0.59 (0.13;2.72)
No	8 (5.3)	142 (94.7)	150 (70.4)		1.00
Symptoms lasted less than 1 week					
Yes	8 (5.8)	130 (94.2)	138 (64.8)	1.06 (0.302)	2.17 (0.47;9.97)
No	2 (2.7)	73 (97.3)	75 (35.2)		1.00
COVID-19 infection in coworkers					
Coworker with COVID-19 in the past year (n = 656)					
Yes	33 (12.3)	236 (87.7)	269 (41.0)	13.97 (0.000)	2.79 (1.59;4.91)
No	17 (4.4)	370 (95.6)	387 (59.0)		1.00
Coworker diagnosed with COVID-19 (n = 269)					
Yes	28 (13.7)	176 (86.3)	204 /(75.8)	1.67 (0.197)	1.78 (0.72;4.43)
No	5 (7.7)	60 (92.3)	65 (24.2)		1.00
Clinical course of COVID-19 in the coworker (n = 259)					
Treated at home	21 (16.2)	109 (83.8)	130 (50.2)	3.03 (0.220)	1.47 (0.66;3.29)
Required hospitalization	5 (7.7)	60 (92.3)	65 (25.1)		0.70 (0.23;2.10)
Visited a physician	7 (10.9)	57 (89.1)	64 (24.7)		1.00
COVID-19 infection in family members in the past year					
Family member with COVID-19 in the past year (n = 656)					
Yes	35 (11.5)	270 (88.3)	305 46.5)	12.02 (0.000)	3.69 (1.49;4.82)
No	15 (4.3)	336 (95.7)	351 (53.5)		1.00
Contact with the infected family member (n = 305)					
Yes	24 (21.8)	86 (78.2)	110 (36.1)	18.11 (0.000)	3.87 (1.97;7.59)
No	11 (5.6)	184 (94.4)	195 (63.9)		1.00
Management of COVID-19 in the infected family member (n = 304)					
Treated at home	19 (9.9)	173 (90.1)	192 (63.2)	12.11 (0.033)	1.04 (0.37;2.90)
Received home-based hospital care	3 (16.7)	15 (83.3)	18 (5.9)		1.75 (0.44;7.04)
Resorted to self-medication	1 (33.3)	2 (66.6)	3 (1.0)		3.50 (0.55;22.31)
Died	3 (8.3)	33 (91.7)	36 (11.8)		0.88 (0.21;3.65)
Did not receive medical care	5 (38.5)	8 (62.5)	13 (4.3)		4.04 (1.27;12.86)
Required hospitalization	4 (9.5)	38 (90.5)	42 (13.8)		1.00
Relationship with the infected family member (n = 305)					
Parent	5 (20.0)	20 (80.0)	25 (8.2)	2.12 (0.547)	2.00 (0.7;5.24)
Sibling	10 (10.5)	85 (89.5)	95 (31.1)		1.05 (0.47;2.37)
Child	9 (12.0)	66 (88.0)	75 (24.6)		1.20 (0.52; 2.75)
Spouse and other	11 (10.0)	99 (90.0)	110 (36.1)		1.00

* Statistically significant associations when p < 0.05.

Workers who reported that their coworkers had been infected with COVID-19 were 1.79 times more likely to receive a diagnosis compared to those who did not report such exposure (PR = 2.79; 95%CI = 1.59-4.91). The prevalence of infection among workers reporting infection in family members was 1.69 greater than among those without such reports (PR = 2.69; 95%CI = 1.49-4.82). Similarly, the prevalence was 2.87 times greater in workers who reported contact with a family member diagnosed with COVID-19 (PR = 3.69; 95%CI = 1.49-4.82), and 3.04 greater among workers whose diagnosed family member did not receive medical care (PR = 4.04; 95%CI = 1.27-12.86) (Table 3).

Increased infection prevalences were observed among workers cohabiting with other families (PR = 1.58); those vaccinated with Moderna (PR = 1.75) or Janssen (PR = 1.24); individuals who did not undergo testing due to lack of symptoms (PR = 0.59); those with symptoms lasting less than 1 week (PR = 2.17); those with diagnosed family members who resorted to self-medication (PR = 3.50); and those with diagnosed family members who were managed with home-based hospital care (PR = 1.75) (Table 3).

### COVID-19 SIGNS AND SYMPTOMS AND COMORBIDITIES ASSOCIATED WITH ITS DIAGNOSIS IN THE WORKING POPULATION UNDER STUDY

Statistically significant associations (p < 0.05) were detected, indicating a higher prevalence of COVID-19 in workers presenting with fever > 38°C (PR = 2.50; 95%CI = 1.39-4.51); dry cough (PR = 2.09; 95%CI = 1.27-3.44); dyspnea (PR = 3.06; 95%CI = 1.89-4.96); myalgia (PR = 2.26; 95%CI = 1.27-4.01); nausea and/or vomiting (PR = 2.07; 95%CI = 1.15-3.72); and abdominal pain (PR = 2.23; 95%CI = 1.01-4.75). Likewise, COVID-19 infection was 1.93 times more prevalent among those reporting dysgeusia (PR = 1.93; 95%CI = 1.17-3.21) and anosmia (PR = 1.72; 95%CI = 1.04-2.84) (Table 4).

**Table 4:** COVID-19 signs and symptoms and comorbidities associated with COVID-19 diagnosis among the working population under study, Medellín, Colombia, 2021 (n = 656)

Characteristic-condition	COVID-19		Total	Chi-square (p-value)^*^	PR (95CI%)
	Yes n (%)	No n (%)	n (%)		
Signs and symptoms of COVID-19 infection and associated comorbidities					
Fever > 38 ºC (n = 297)					
Yes	37 (23.4)	121 (76.6)	158 (53.2)	10.45 (0.001)	2.50 (1.39;4.51)
No	13 (9.4)	126 (90.6)	139 (46.8)		1.00
Dry cough (n = 297)					
Yes	22 (27.2)	59 (72.8)	81 (27.3)	8.48 (0.003)	2.09 (1.27;3.44)
No	28 (13.0)	188 (87.0)	216 (72.7)		1.00
Dyspnea (n = 297)					
Yes	20 (37.7)	33 (62.3)	53 (17.9)	20.12 (0.000)	3.06 (1.89;4.96)
No	30 (12.3)	214 (87.7)	244 (82.1)		1.00
Sore throat (n = 297)					
Yes	7 (25.0)	21 (75.0)	28 (9.4)	1.47 (0.225)	1.56 (0.77;3.14)
No	43 (16.0)	226 (84.0)	269 (90.6)		1.00
Myalgia (n = 297)					
Yes	36 (22.8)	122 (77.2)	158 (53.2)	8.54 (0.003)	2.26 (1.27;4.01)
No	14 (10.1)	125 (89.9)	139 (46.8)		1.00
Adynamia (n = 297)					
Yes	7 (28.0)	18 (72.0)	25 (8.4)	2.43 (0.119)	1.77 (0.89;3.51)
No	43 (15.8)	229 (84.2)	272 (91.6)		1.00
Headache (n = 297)					
Yes	23 (19.3)	96 (80.7)	119 (40.1)	0.88 (0.347)	1.27 (0.76;2.11)
No	27 (15.2)	151 (84.8)	178 (59.9)		1.00
Chest pain (n = 297)					
Yes	4 (28.6)	10 (71.4)	14 (4.7)	0.69 (0.402)	1.76 (0.73;4.19)
No	46 (16.3)	237 (83.7)	283 (95.3)		1.00
Diarrhea (n = 297)					
Yes	13 (24.5)	40 (75.5)	53 (17.8)	2.72 (0.098)	1.62 (0.92;2.82)
No	37 (15.2)	207 (84.8)	244 (82.2)		1.00
Nausea and/or vomiting (n = 297)					
Yes	10 (31.3)	22 (68.7)	32 (10.8)	5.32 (0.021)	2.07 (1.15;3.72)
No	40 (15.1)	225 (84.9)	265 (89.2)		1.00
Abdominal pain (n = 296)					
Yes	5 (35.7)	9 (64.3)	14 (4.7)	3.70 (0.054)	2.23 (1.01;4.75)
No	45 (16.0)	237 (84.0)	282 (95.3)		1.00
Ageusia/dysgeusia (n = 297)					
Yes	27 (24.1)	85 (75.9)	112 (37.7)	6.79 (0.009)	1.93 (1.17;3.21)
No	23 (12.4)	162 (87.6)	185 (62.3)		1.00
Anosmia (n = 297)					
Yes	25 (23.1)	84 (77.8)	108 (36.5)	4.57 (0.032)	1.72 (1.04;2.84)
No	25 (13.3)	163 (86.7)	188 (63.5)		1.00
Visited a physician for any of the previous symptoms (n = 206)					
Yes	43 (60.6)	28 (39.4)	71 (34.5)	84.42 (0.000)	20.4 (7.64;54.60)
No	4 (3.0)	131 (97.0)	135 (65.5)		1.00
No COVID-19 symptoms in the past year (n = 296)					
Yes	8 (8.7)	84 (91.3)	92 (31.1)	6.38 (0.011)	0.42 (0.20;0.86)
No	42 (20.6)	162 (79.4)	204 (68.9)		1.00
Underwent testing for any of the previous symptoms (n = 120)					
Yes	40 (70.2)	17 (29.8)	57 (47.5)	36.3 (0.000)	4.40 (2.44;8.00)
No	10 (15.9)	53 (84.1)	63 (52.5)		1.00
Comorbidities associated with COVID-19 infection					
Arterial hypertension (n = 656)					
Yes	17 (10.7)	142 (89.3)	159 (24.2)	2.80 (0.093)	1.61 (0.90;2.80)
No	33 (6.6)	464 (93.4)	497 (75.8)		1.00
Diabetes (n = 656)					
Yes	11 (16.2)	57 (83.8)	68 (10.4)	7.88 (0.005)	2.43 (1.31;4.53)
No	39 (6.6)	549 (93.4)	588 (89.6)		1.00
Chronic obstructive pulmonary disease (n = 656)					
Yes	2 (16.7)	10 (8.3)	12 (1.8)	1.42 (0.233)	2.23 (0.61;8.15)
No	48 (7.5)	596 (92.5)	644 (98.2)		1.00
Depressive symptoms (n = 656)					
Yes	2 (7.1)	26 (92.9)	28 (4.3)	0.07 (0.922)	0.93 (0.23;3.65)
No	48 (7.6)	580 (92.4)	628 (95.7)		1.0
Obesity (n = 656)					
Yes	18 (9.5)	171 (90.5)	189 (28.4)	1.28 (0.257)	1.37 (0.79; 2.38)
No	33 (6.9)	443 (93.1)	476 (71.6)		1.00

* Statistically significant associations when p < 0.05.

The probability of receiving a COVID-19 diagnosis was significantly higher (p < 0.05) in individuals who sought care for any signs and symptoms (PR = 20.4; 95%CI = 7.64-54.6) and in those who underwent diagnostic testing (PR = 4.40; 95%CI = 2.44-8.00) (Table 4).

Finally, a significant association (p < 0.05) was identified between COVID-19 diagnosis and presence of diabetes (PR = 2.43; 95%CI = 1.31-4.53). While not reaching statistical significance, infection prevalence was 61.0% greater among participants with hypertension, 1.23 greater among those diagnosed with COPD, and 37.0% greater among individuals with obesity (Table 4).

### CONDITIONS THAT HELP EXPLAIN COVID-19 DIAGNOSIS IN THE WORKING POPULATION UNDER STUDY

Significant associations (p < 0.05) were observed when examining conditions that jointly contributed an increased proportion of COVID-19 diagnosis, including contact with an infected family member in the past year (adjusted prevalence ratio [APR] = 2.72; 95%CI = 1.02-7.23), and the infected family member not having received medical care (APR = 19.50; 95%CI = 1.09-348.33). These associations remained significant after adjusting for sex, cohabitation with other families, contact with a probable or confirmed case of COVID-19, infected coworkers, infected family members, management received by coworkers or family members, and contact with an infected family member in the past year (Table 5).

**Table 5:** Conditions that contribute to explain COVID-19 diagnosis among the working population under study, Medellín, Colombia, 2021

Condition - characteristic	CPR	95%CI		APR	95%CI	
		LT	UT		LT	UT
Model 1. Sociodemographic conditions and infection characteristics						
Age (years) (Ref: 18-29)	1.41	0.91	2.23	0.99	0.78	1.26
30-44	0.84	0.19	3.62	4.76	0.35	64.25
45-59	0.89	0.22	3.58	0.62	0.15	3.25
≥ 60	0.98	0.24	3.99	1.38	0.38	5.02
Sex (Ref: Male)	0.77	0.45	1.32	2.15	0.74	6.25
Cohabitation with other family (Ref: Yes)	1.58	0.84	2.99	0.47	0.04	1.66
Contact with a probable or confirmed case of COVID-19 (Ref: Yes)	3.23	1.91	5.47	2.42	0.73	8.02
Coworker was diagnosed with COVID-19 in the past year (Ref: No)						
Yes	1.78	0.72	4.43	2.75	0.60	12.66
Management of coworker infected with COVID-19 (Ref: Visited a physician)						
Treated at home	1.47	0.63	3.29	2.23	0.59	8.45
Required hospitalization	0.70	0.23	2.10	1.19	0.27	5.33
Contact with a family member infected with COVID-19 in the past year (Ref: No)						
Yes	3.87	1.97	7.59	2.72	1.02	7.23
Management of COVID-19 in the family member (Ref: Required hospitalization)						
Did not receive care	4.04	1.27	12.86	19.50	1.09	348.33
Treated at home	1.04	0.37	2.90	0.90	0.17	4.77
Receive hospital-based home care	1.75	0.44	7.04	2.40	0.31	18.72
Resorted to self-medication	3.50	0.55	22.31	0.28	0.00	NC
Died	0.88	0.21	3.65	0.00	0.00	NC
Model 2. Symptoms and comorbidities						
Sex (Ref: Men)	0.77	0.45	1.32	1.09	0.29	4.13
Fever > 38 °C (Ref: Yes)	2.50	1.39	4.51	0.21	0.02	1.96
Dry cough (Ref: Yes)	2.09	1.27	3.44	0.53	0.12	2.40
Dyspnea (Ref: Yes)	3.06	1.89	4.96	3.69	0.82	16.67
Myalgias (Ref: Yes)	2.26	1.27	4.01	0.70	0.06	8.38
Diarrhea (Ref: Yes)	1.62	0.92	2.82	0.70	0.14	3.45
Nausea and/or vomiting (Ref: Yes)	2.07	1.15	3.72	0.52	0.07	4.00
Abdominal pain (Ref: Yes)	2.23	1.01	4.75	0.20	0.02	2.51
Ageusia/dysgeusia (Ref: Yes)	1.93	1.17	3.21	2.65	0.35	20.01
Anosmia (Ref: Yes)	1.72	1.04	2.84	0.73	0.13	4.18
Consulted the physician for any of the symptoms (Cr Yes)	20.40	7.64	54.6	126.37	14.12	1,130.88
Diabetes (Ref: Yes)	2.43	1.31	4.53	1.40	0.32	1.85
Chronic obstructive pulmonary disease (Ref: Yes)	2.23	0.61	8.15	5.29	0.25	113.64
Arterial hypertension (Ref: Yes)	1.61	0.90	2.80	0.38	0.08	185.26

APR = adjusted prevalence ratio; CPR = crude prevalence ratio; LT = lower threshold; NC = not calculated; Ref = category of reference; UT = upper threshold.

While not statistically significant, age demonstrated a shift from being linked to lower infection prevalence to higher prevalence, particularly among individuals aged 30-44 years (APR = 4.76) and ≥ 60 years (APR = 1.38), after adjusting for the other variables included in the analysis. Similarly, male sex was associated with higher infection prevalence (APR = 2.15) after adjusting for the other variables. Increased infection prevalence was also observed in participants who had contact with a confirmed case (APR = 2.42), had a diagnosed coworker (APR = 2.75), whose infected coworker received home-based care (APR = 2.23), and whose infected family member required home hospitalization (APR = 2.40) (Table 5).

Regarding symptoms and comorbidities, consulting for any of the reported symptoms was associated with a higher prevalence of COVID-19 (p < 0.05) (APR = 126.37. 95%CI = 14.12-1,130.88), after adjusting the other variables included in the analysis. While not reaching statistical significance, increased infection prevalence was also associated with dyspnea (APR = 3.69), ageusia/dysgeusia (APR = 2.65), and diagnosis of diabetes (APR = 1.40) and COPD (APR = 5.29) (Table 5).

## DISCUSSION

The present study revealed intrinsic factors within the analyzed working population that could have positioned them as a group at higher risk for adverse health outcomes. Among these factors were older age, with most individuals in early adulthood and a smaller proportion in late adulthood, consistent with the findings of Garzón Duque et al.^8^

Older age has been consistently observed in multiple studies. A study examining health indicators and conditions in street vendors revealed evidence of lack of social protection in older age. Of the 170 workers randomly selected from a population of 422 informal workers in central Medellín, only five were younger than 25 years, whereas 82.0% were over 35 years in 2009.^9^ This proportion of individuals under 25 years compared with those over 40 has been increasing, as demonstrated by other studies.^2^,^10^-^12^

COVID-19 emerged as global emergency, underscoring the importance of identifying associated with infection. Systematic reviews and meta-analysis conducted in Latin America countries, such as Cuba, concluded that the overall risk of severe infection was 5.60 for patients with chronic kidney disease, 4.05 for those with arterial hypertension, and 3.53 for those with diabetes.^13^ In the present study, an associated was found between COVID-19 diagnosis and presence of diabetes, with a 1.43-fold higher prevalence among workers with this condition. While not reaching statistical significance, infection prevalence was 61.0% higher in individuals with hypertension, 1.23 time higher in those with COPD, and 37.0% in those with obesity; however, these findings did not specifically apply to patients who developed severe infection.

The study population had previously participated in research examining multimorbidity among older adult workers, identifying work-related conditions that increase the prevalence of chronic diseases. A significant association was observed in workers with more than 5 years of experience in the sector, and those exposed to chemical substances exhibited higher rates of multimorbidity. In a previous study conducted in Medellín, Colombia, 63.0% of older adult street vendors reported at least one chronic disease, and 62.0% presented multimorbidity.^8^ The most prevalent conditions were: diabetes mellitus (25.8%), overweight/obesity (14.9%), arterial hypertension (64.9%), and moderate/severe depressive symptoms (14.4%). These conditions increased socio-environmental and occupational vulnerability, thereby exacerbating health effects during the COVID-19 pandemic.^8^

Diabetes is common in the study population. A study conducted in Medellín demonstrated a significant association between obesity and diabetes in workers engaged in subsistence jobs. It was found that 27.4% of participants were obese and 10.4% had diabetes, with diabetes showing a significant association with COVID-19, particularly impacting individuals with both diabetes and hypertension.^14^

A study on hypertension revealed that 32.4% of workers consumed fried foods, one in four reported a sedentary lifestyle, and 16.8% had low physical activity levels.^2^ Another study identified factors associated with hypertension, concluding that older age, female sex, Afro-Colombian ethnicity, belonging to socioeconomic stratum zero, moderate food insecurity, diabetes, and heart disease were all associated with arterial hypertension.^15^

The findings of this study indicate that COVID-19 diagnosis was more prevalent among workers who had contact with an infected family member over the past year, particularly when the latter did not receive medical care. Moreover, infection prevalence was lower in younger workers. Increased disease prevalence was observed in individuals presenting with symptoms such as dyspnea and ageusia, and in those with pre-existing diabetes or COPD.

COVID-19 appears to have affected how the evaluated working population sought health care, since a higher percentage did not seek medical care if their symptoms lasted less than 1 week. The most common symptoms were fever, myalgia, headache, ageusia, anosmia, dry cough, and, less frequently, dyspnea. Nearly all participants (99.5%) reported not knowing where to obtain a diagnostic test, and only a small proportion actually underwent testing. In addition, a high percentage of their family members had COVID-19, although most were treated at home without confirmation of diagnosis.

Furthermore, recent studies in Medellín reported findings consistent with the present research. A study published in 2022 found that most subsistence supported themselves and two or more dependents and generally lived in overcrowded conditions, a situation that worsened during the pandemic due to loss of income. That year, 83.5% of workers earned below the minimum wage. The most affected population groups included female heads of household, and the most prevalent needs were food, housing rent, and payment of public utilities.^16^

The main limitation of this study is that it was conducted exclusively with a group of workers in Medellín. Therefore, caution is needed when comparing its results with those of other local and national studies, since other populations may have distinct physical and socioeconomic characteristics that hinder detailed comparisons.

Although selection bias was minimized through the strict application of inclusion criteria. While this approach may reflect certain characteristics of the study population, it does not allow for the measurement of inference error and is therefore less robust. Nevertheless, it is considered more suitable than a non-representative sampling strategy, which can only be applied to the data collection sample, and was particularly appropriate during the period of mandatory lockdowns and staggered quarantines.

The inclusion criteria required workers to have been in their occupation for at least 3 years, to ensure that the work predated the pandemic and to avoid including those who have shifted from other sectors or started informal street work as a result of the pandemic. Due to its design, this study was not intended to establish causal relationships and associations; therefore, no interpretations or analyses are made in this regard. Rather, the study focused on identifying relationships and associations between signs, symptoms, comorbidities, and COVID-19 infection in diagnosed workers. Future research in similar populations under exceptional circumstances such as pandemics, wars, or environmental or disaster-related emergencies, would benefit from longitudinal designs that could allow, when necessary and feasible, for causal inferences.

Considering the above, similar results were observed in Bogotá and Medellín. Informal workers had limited knowledge of occupational health and safety; however, due to a lack of job opportunities, such work was considered the only option to meet workers’ personal and family obligations. Throat disorders and COVID-19 infection were reported as the most frequent illnesses in this population, which is inherently more vulnerable due to the absence of mechanisms to protect their income during the pandemic-induced economic halt.^17^,^18^ Therefore, as highlighted by the ILO, epidemics and economic crises disproportionally affect disadvantaged groups, exponentially increasing “alternative” forms of work, such as informal employment. The most impacted groups include older and younger people, who have fewer job opportunities, as well as migrants, who also lack income compensation mechanisms.^19^

In May 2020, the World Health Organization described vulnerability factors predisposing individuals to COVID-19 infection, such as overcrowding, dependence on informal employment and minimum wages, limited access to health care, food insecurity, malnutrition, marginalized and underserved communities, among others. A study in Mexico showed an association between vulnerability factors and risk factors, demonstrating a cause-and-effect relationship that, as a consequence, increases susceptibility to COVID-19 infection.^20^

In the present study population, 15.0% lived with other families in the mandatory isolation period, and 86.2% of their symptomatic family members were diagnosed with COVID-19. Even though 97.0% of workers were covered by a health system, 96.3% had not visited a physician in the past year, reflecting the previously mentioned risk factors associated with SARS-CoV-2 infection.

## CONCLUSIONS

In response to the research question, the study found that workers with subsistence jobs on streets and sidewalks were affected in their own health and that of their families during the pandemic in 2021, particularly those with pre-existing comorbidities and socioeconomic and work conditions. Therefore, it is essential to consider that the socioeconomic and occupational vulnerability of this population increases during global crises such as a pandemic, underlining the need for public policies that strengthen their preparedness and ability to respond at individual, family and community levels to future contingencies similar to that of 2020, enabling them to cope with and withstand environmental and human-made disasters in a more informed manner, provided that they have sufficient resources and opportunities.
